# Co-occurring Hearing Loss and Cognitive Decline in Older Adults: A Dual Group-Based Trajectory Modeling Approach

**DOI:** 10.3389/fnagi.2021.794787

**Published:** 2021-12-24

**Authors:** Yvonne Tran, Diana Tang, Charles Lo, Catherine McMahon, Paul Mitchell, Bamini Gopinath

**Affiliations:** ^1^MU Hearing, Department of Linguistics, Macquarie University, Sydney, NSW, Australia; ^2^Management Disciplinary Group, Wentworth Institute for Higher Education, Sydney, NSW, Australia; ^3^Centre for Vision Research, Department of Ophthalmology and Westmead Institute for Medical Research, University of Sydney, Sydney, NSW, Australia

**Keywords:** hearing loss, cognitve function, aging, older adults, group-based trajectory model, dual trajectory models, blue mountains hearing study

## Abstract

Hearing loss and cognitive impairments are both highly prevalent neurological complications for older adults. While there is growing evidence to suggest that these two conditions are interrelated, little research has been conducted that directly examines the progression and developmental trajectories of these complications contemporaneously. The aim of the study is to identify the distinct trajectory profiles for hearing loss and cognitive function in an older population over a 10-year period. Through dual trajectory modeling, the interrelationship, co-occurring movements, and overlaps between these two complications were examined. We also investigated the influence of hearing aid ownership on cognitive function trajectories. We utilized longitudinal data from 1,445 participants in the Blue Mountains Hearing Study (aged 55+ years) involving repeated measures from a population-based survey with audiometric hearing assessments. Cognitive function was assessed using the Mini-Mental State Examination (MMSE). The group-based trajectory modeling (GBTM) identified three trajectory profiles for both hearing loss and cognitive function in two older age groups (55–69 years and 70+ years). The outputs from the dual trajectories models showed the conditional probability for “no hearing loss” trajectories to be around 90% more likely to have “high-normal” cognitive function, demonstrating co-occurring overlap. In contrast, for “moderate to severe hearing loss” trajectories, the conditional probability drops to 65% and 79% for the 55–69 age group and 70+ age group respectively. There was also an increasing probability for “cognitive decline” conditional on the severity of hearing loss with 6.7%, 7.5%, and 8.7% for no hearing loss, mild hearing loss, and moderate to severe hearing loss trajectory groups. While we did not find any statistically significant difference in the influence of hearing aid use in the cognitive function trajectories, there was a consistent greater representation of non-hearing aid users in the trajectories with poorer cognitive function. This study found GBTM to identify trajectories that were in agreement with our current understanding of hearing loss and cognitive impairment in older adults. This study also adds to the existing evidence-base as dual trajectories demonstrated co-occurrence in developmental changes in these two common neurological complications for the older population.

## Introduction

Growth in the older population is increasing worldwide mostly due to the rise in life expectancy. It has been estimated that by 2050 the number of persons aged 65 years or over will double to 1.5 billion (United Nations, Department of Economics and Social Affairs, Population Division, 2019). With this rapid demographic aging, the prevalence of disease and disability will likely surge. This is concerning for highly prevalent neurological complications such as age-related hearing loss and cognitive impairment. The World Health Organization (WHO) has projected that by 2050, one in four people will have some degree of hearing loss (World Health Organization, 2021), with age-related hearing loss being most prevalent (Yang et al., 2015). Similarly, the projected rise for dementia, a syndrome that leads to deterioration of cognitive function, is estimated to reach 139 million in 2050, which is, 2.5 times greater than the current prevalence (World Health Organization, 2017 ). Aside from both being highly prevalent, there is also growing evidence to suggest that these two conditions are interrelated, possibly leading to a compounding burden (Nadhimi and Llano, 2021). Studies are showing hearing loss to be significantly associated with cognitive decline and increases the risk for cognitive impairment (Loughrey et al., 2018). One postulation for their linkage is that hearing loss has associations with language comprehension and that cognitive decline will increase the contributing effect of hearing loss as a cognitive impairing factor (Peracino and Pecorelli, 2016).

In the last decade, there has been an increase in research addressing the associations between cognitive impairment and hearing loss. In the Baltimore Longitudinal Study of Aging (BLSA), a greater hearing loss was found to be associated with poorer cognitive function with lower scores on measured mental status (Mini-Mental State Exam), memory (Free Recall), and executive function (Stroop Mixed, Trail Making B; Lin et al., 2011a). A meta-analysis from 36 unique studies on cognitive function and hearing loss also found significant associations in 10 cognitive domains in cross-sectional studies including executive function, episodic and semantic memory, and visuospatial ability (Loughrey et al., 2018). In addition to hearing loss being associated with poor cognitive function, it is also found to be a risk factor for cognitive impairment and dementia (Lin et al., 2011b; Deal et al., 2017; Livingston et al., 2020) with possible brain bases for this relationship (Griffiths et al., 2020; Slade et al., 2020). In another meta-analysis, a synthesis of four cohort studies found a risk ratio of 1.3 times higher risk for mild cognitive impairments with hearing loss, and a synthesis of seven cohort studies found a risk ratio of 2.4 times higher risk for dementia for hearing impaired (Wei et al., 2017).

For people who experience age-related hearing loss the main clinical intervention for mild to moderate hearing loss is the provision of hearing aids (Ferguson et al., 2017). Some studies have examined whether the use of hearing aids could help mitigate cognitive decline. Estimated cognitive decline in memory and global function was found to be greatest in participants with moderate hearing loss who did not wear a hearing aid (Deal et al., 2015). There was also support that rates of cognitive decline in hearing aid users were not significantly different to those with no hearing loss but significantly higher in non-hearing aid users (Amieva et al., 2015). Dawes et al. (2015) have also found support for hearing aid use showing associations with better cognition. They posit that hearing aid use may boost self-efficacy which then impacts cognitive performance (Dawes et al., 2015).

There has also been some research focused on the progression and developmental trajectories of hearing loss and cognitive function. Trajectories describe the evolution of a behavior or biomarker over time (Elmer et al., 2018). Longitudinal analysis using linear mixed-effects modeling (LMM) has been used to show steeper declines in brain volume in temporal lobe structures with hearing impairment (Armstrong et al., 2019). Similarly, using LMM, a faster decline in mini-mental state scores was found for hearing loss vs. no hearing loss groups (Gurgel et al., 2014). Using group-based trajectory modeling (GBTM), Ferraro et al. (2021) examined three outcomes, cognitive, physical function, and disability from the Activities of Daily Living (ADL) score and identified group profiles. Utilizing GBTM they found three trajectory groups for these outcomes, which were labeled as “good”, “intermediate, ” and “severe”. The “severe” trajectory group was found to have the worst performance with a decline in all the three outcomes and highest incidence of dementia (Ferraro et al., 2021). While LMM can be used to map prospective changes, group differences are pre-determined from measured cut-offs, that is, the existence of distinct developmental trajectories is assumed* a priori*. This means LMM cannot be used to test for the presence of distinct trajectories. Using finite mixture modeling, GBTM maximizes the information available in multivariate longitudinal data to track the course of an outcome and assess the heterogeneity in the population allowing for a more precise individual classifications into various groups that comprise the taxonomy (Nagin and Odgers, 2010). It allows for the identification of meaningful subgroups and the frequency distribution in each subgroup within a population that are not based on a measured set of characteristics (Nagin, 2014), that is, rather than assuming existing trajectories* a priori*, GBTM allows the trajectories to emerge from the data itself and determines the form and number of groups that best fit the data (Nagin, 1999). For this current study, profiling developmental pathways will contribute to our understanding of the relationship between types of hearing loss and types of cognitive function.

Using an extension of the GBTM, a dual trajectory approach will be used to examine how hearing loss and cognitive impairment trajectories co-occur and evolve contemporaneously over time. “Dual disorders” are common in health research and dual trajectory models provide an opportunity to study this phenomenon. Previous studies have found that concurrent sensory impairment such as vision and hearing loss increases the risk of dementia (Kuo et al., 2021b). Gopinath et al. (2013) have found dual sensory impairment to be associated with increased mortality with a greater risk of death than that of vision loss or hearing loss alone. This demonstrates that concurrent impairments often compound the burden and additional resources are required to reduce risks (Kuo et al., 2021b). This study will examine the interrelationship between hearing loss and cognitive impairment simultaneously. By examining the interrelationship across various trajectories, the dual models will allow us to understand the multidimensional and dynamic association between hearing loss and cognitive impairment. The aim of this novel epidemiological study is to identify the distinct trajectories and profiles for hearing loss and cognitive function in an older population over a 10-year period. Through the application of a dual-GBTM, the evolution of developmental trajectories from hearing loss and cognitive function will be examined contemporaneously. A secondary aim is to investigate the influence of hearing aid use on cognitive function trajectories, to examine whether hearing aid use mitigates poorer cognitive outcomes.

## Materials and Methods

### Study Population

The study population was from the Blue Mountains Cohort Study, which examined the long-term development and progression of sensory problems such as vision and hearing loss. We utilized the hearing study data which collected demographic, lifestyle factors, health outcomes, and audiometric measures from 1997 (baseline), with 5- and 10-year follow-up to 2009 (Gopinath et al., 2009). The Blue Mountains Hearing Study (BMHS) is a population-based survey of older adults (ages 50+) from the Blue Mountains area, west of Sydney, Australia. For the current study, we examined data from 1,445 participants from the BMHS, where data from at least two time-points for both pure-tone audiometry and survey responses on cognitive function were available. The BMHS was conducted in accordance with the Declaration of Helsinki and was approved by The University of Sydney Human Research Ethics Committee (Reference: HREC 9826).

### Sub-group Analyses

The BLSA examined longitudinal phenotypic dimensions of aging and found changes are not necessarily linear, as an example, differences between 30 and 50 years are not equivalent to that of 60 and 80 years (Kuo et al., 2020), supporting age-stratified analysis. As increasing age is likely to influence the results of developmental trajectories, with greater hearing loss and cognitive decline in older respondents (Hong et al., 2016), we stratified the study population into two groups. We chose a cut-off of age 70 as it has previously been established that cognitive function starts declining after this age and brain white matter volume is also known to decrease more rapidly and ventricular volume is known to increase rapidly at this age (Harada et al., 2013; Kuo et al., 2020). The two age subgroups examined for the current study were those with age ranges 55–69 and those that were age 70+. There were 847 participants between 55 and 69 years of age and 598 participants over 70 years of age.

In addition, as we were interested in the influence of hearing aid use on the co-occurring developmental trajectories for hearing loss and cognitive decline, we conducted subgroup analysis with the hearing aid owners. As the number of hearing aid owners is substantially smaller there is an overall group imbalance, to adjust for the imbalance a matched comparison group was used. We investigated differences in cognitive function between a hearing aid group vs. a non-hearing aid group.

### Measures

#### Audiometric Testing

To obtain an audiometric measure of hearing loss, pure-tone audiometry was administered by audiologists in sound-treated facilities. The air conduction thresholds were obtained using a Madsen OB822 audiometer (Madsen Electronics Copenhagen, Denmark) calibrated to Australian standards. Stimuli were presented through supra-aural headphones using standard TDH-39 earphones. Audiometric thresholds for air-conduction stimuli in both ears were established for frequencies at 0.25–8.0 kHz. For a measure of hearing loss, we used the conventional hearing status in accordance with the World Health Organization (WHO), using averaged pure-tone audiometric hearing thresholds at 0.5, 1, 2, and 4 kHz. A four-frequency pure tone average PTA (dB HL) greater than 25 dB HL in the better hearing ear was used to define hearing loss. This defines hearing loss as bilateral. Hearing loss was defined as mild at 25–45 dB, and moderate to severe at greater than 45 dB (Chia et al., 2007).

#### Mini-Mental State Examination (MMSE)

Cognitive function was assessed using the mini-mental state examination (MMSE), often used as a check for cognitive impairment (Folstein et al., 1975). MMSE consists of 11 main items that assess attention and orientation, memory, registration, recall, calculation, language, and ability to draw a complex polygon (Folstein et al., 1975). MMSE has a possible scores range of 1 to a maximum of 30. Questions are scored with 0 for incorrect and 1 for correct responses. Within each item are sub-scores, for example, the question “What is the date today” has a possible 3-points for the correct day (1 point), the correct month (1 point), and the correct year (1 point). Scores between 25 and 30 indicate normal cognitive function. MMSE is a validated screen for dementia with a score of less than or equal to 24 used as the cut-off for cognitive impairment or dementia (Creavin et al., 2016).

#### Hearing Aid Question

To obtain information regarding HA use in the study population we used the question “Do you or have you ever worn a hearing aid?” from the BMHS survey. Response for this question was categorized as “Yes” or “No”. HA owners were defined as those reporting “Yes” that they have ever worn a HA, and conversely non-HA owners are those that responded “No”.

### Group-Based Trajectory Modeling (GBTM)

To identify developmental trajectories for hearing loss and cognitive function, group-based trajectory modeling (GBTM) was used. GBTM is an exploratory technique used for isolating developmental trajectories within a population with the goal of identifying subgroups that follow distinct trajectories over time (Nagin, 2014). It was used to classify and identify the number of unobserved heterogeneities in this sample of older adults for hearing loss and cognitive function. To guide the choice for the optimal number of trajectories we evaluated the fit for each number of classes, using the fit indices from information criterion indices Akaike information criterion (AIC) and Bayesian information criterion (BIC), a measure of entropy, Lo-Mendell-Rubin (LMR) test and the parametric bootstrap likelihood-ratio test (BLRT) and interpretability of the model (Nylund et al., 2007). The chosen number of classes/trajectories were determined by the change in the rate of decrease in the information criterion, a higher value of entropy indicating how well the classification distinguishes from one group to another, and a significant LMR test or BLRT likelihood-ratio test showing whether the number of classes was a better fit than the number of classes prior, as well as interpretability of the trajectories.

To examine the co-occurring relationship between hearing loss and cognitive function trajectories we used the extension of the GBTM, the dual trajectory analysis (Jones and Nagin, 2007). The dual-GBTM is a multivariate version of the GBTM and estimates the joint developmental trajectories of two distinct but related co-occurring longitudinal outcomes (Xie et al., 2010). The evolving contemporaneous associations between hearing loss and cognitive function trajectories were measured as the conditional probability of membership in each of the trajectories from the dual measurement time series. Both GBTM and dual-GBTM were conducted in Mplus version 7.3 (Muthen and Muthen, 1998–2011).

### Statistical Analysis

Statistical analyses were conducted using SPSS version 27 (IBM Corp, 2020). Descriptive statistics were calculated as means with standard deviations or frequencies and percentages. T-tests and chi-square tests were used to compare demographic, hearing loss characteristics, and cognitive function between the 55 and 69 age group and the 70+ age group. The Chi-square test was also used to examine the differences in proportion between hearing aid users and non-hearing aid users in the cognitive function trajectories. We matched the hearing group owners with a group similar in age, sex, and hearing loss levels using propensity score matching. Propensity score matching was performed in R (Version 4.3) using the R package MatchIt (Ho et al., 2007). Propensity scores were calculated using logistic regression and matching was conducted with the nearest neighbor approach using the optimal method. Age, sex, and baseline PTA (dB HL) were entered into the logistic regression model. Standardized mean differences (SMD) and variance ratios were used to assess covariate balance diagnostics after matching. A cut-off of SMD ≤ 0.2 and variance ratio close to 1 and <2 shows covariate balance and good matching (Zhang et al., 2019).

## Results

Table 1 shows the descriptive statistics for the two age groups. There were significant differences between the two age groups for hearing loss characteristics, cognitive function, and hearing aid ownership. Both groups showed a slight decline in cognitive function and greater increases in hearing loss over time. There were greater hearing aid users in the older age group of 70+ years.

**Table 1:** Demographic, cognitive function, and hearing loss characteristics for the two age groups in the study.

Characteristics	Age group 54–69	Age group 70+	*p*-value
	*N* = 847	*N* = 598	
Age, Mean (SD)	62.7 (4.1)	75.4 (4.3)	<0.001
Sex, N (%)	Male: 369 (43.6)	Male: 244 (40.8)	0.295
	Female: 478 (56.4)	Female: 354 (59.2)	
Baseline PTA (dB HL), Mean (SD)	18.2 (12.0)	27.5 (12.6)	<0.001
5-years PTA (dB HL), Mean (SD)	20.5 (12.4)	31.8 (13.4)	<0.001
10 years PTA (dB HL), Mean (SD)	26.6 (13.6)	37.7 (15.6)	<0.001
Cognitive function (MMSE) Baseline, Mean (SD)	28.8 (1.5)	28.4 (1.9)	<0.001
Cognitive function (MMSE) 5-years, Mean (SD)	28.9 (1.7)	28.2 (2.5)	<0.001
Cognitive function (MMSE) 10-years, Mean (SD)	27.9 (2.7)	26.7 (3.5)	<0.001
Hearing Aid Use, N (%)	Yes: 42 (5)	Yes: 110 (18.4)	<0.001
	No: 805 (95)	No: 488 (81.6)	

*Abbreviations: HL, Hearing loss; PTA, Pure-tone average; db, decibels; SD, Standard deviation; MMSE, Mini-Mental State Examination*.

### Hearing Loss and Cognitive Function Trajectories for the 55–69 Years Age Group

**Figure 1:**
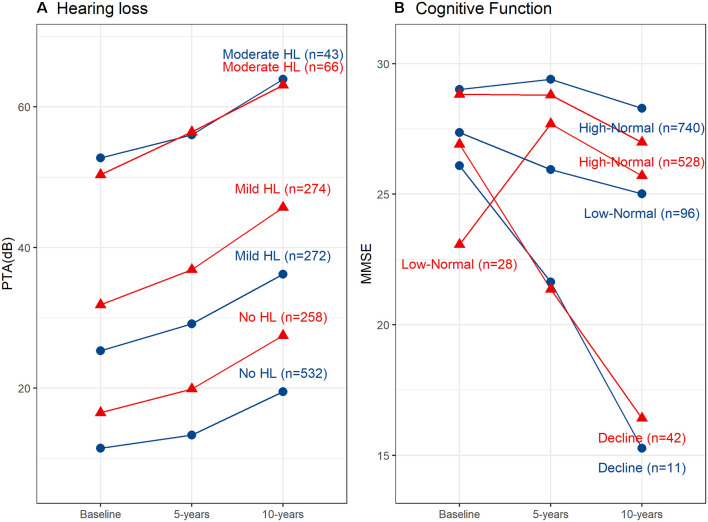
Hearing loss and cognitive function trajectories for the two age groups. **(A)** Trajectories for any hearing loss PTA (dB HL). Age group 55–69 years shown in red and age group 70+ years shown in blue. **(B)** Trajectories for cognitive function measured using the Mini-Mental State Examination (MMSE). Age group 55–69 years shown in red and age group 70+ years shown in blue. HL, Hearing loss; PTA, Pure-tone average; db, decibels.

Fit indices determined that three trajectories were the best solution for both hearing loss and cognitive function (see Supplementary Material for details). Figure 1A shows the hearing loss and cognitive function trajectories for the 55–65 age group. Table 2 and Table 3 show the descriptive statistics for the hearing loss and cognitive function trajectories for this group. Age was found to be significantly different in the three trajectories with increasing age for increasing hearing loss (*F*_(2, 844)_ = 46.13, *p* < 0.001). There were also significant sex differences between the three trajectories (*χ*^2^ = 12.6, *p* = 0.002), with a larger proportion of females in the no hearing loss trajectory and a smaller proportion in the moderate hearing loss trajectory. The three classes identified from GBTM for hearing loss were labeled as no hearing loss, mild hearing loss, and moderate to severe hearing loss. This was named based upon their baseline hearing loss status since hearing loss is considered as a status from a specific timepoint rather than over a period of time. The mean (95% CI) of any hearing loss for the three trajectories were 14.8 (14.3–15.3), 30.2 (29.6–30.9), and 57.6 (55.9–59.3) dB HL. Hearing loss was found to increase over time in all three trajectories over the 10-year period with an average rate of change in the three trajectories being 1 dB hearing loss per year. For the 55–65 age group, 62.8% belonged to the trajectory with no hearing loss, 32.0% belonged to a trajectory with mild hearing loss, and only 5.2% of the group in the moderate to severe hearing loss trajectory. There were three cognitive function trajectories labeled as a high-normal cognitive function, low-normal cognitive function, and declining cognitive function. The high-normal cognitive trajectory stayed within the normal MMSE range for the 10-year period. The low-normal cognitive trajectory showed a decline towards mild cognitive impairment by 10 years, whereas the declining cognitive function trajectory showed cognitive impairment at both 5- and 10-year follow-ups (see Figure 1). For the three cognitive function trajectories, age was just significantly different (*F*_(2, 844)_ = 3.11, *p* = 0.045) between the trajectories. Similar to the hearing loss trajectories there were differences in the proportion of males and females for the cognitive function trajectories (*χ*^2^ = 14.7, *p* < 0.001). There was a smaller proportion of females in the low-normal and decline trajectories. The rate of change for the declining trajectory is at 1.1 MMSE points per year. The mean (95% CI) MMSE scores for these three trajectories were 28.9 (28.8–29.0), 26.1 (25.9–26.3), and 21.0 (20.2–21.8). For this age group, 86.9% of this cohort belonged to a stable high-normal cognitive function trajectory, 11.7% in the stable low-normal cognitive function trajectory, and only 1.4% belonged to a trajectory with declining cognitive function.

**Table 2:** Descriptive statistics for hearing loss trajectories.

Sub-group	Characteristics	No HL	Mild HL	Moderate HL
		Mean (SD)	Mean (SD)	Mean (SD)
Age 55–69	N=	532	272	43
Hearing Loss	
	Age	61.71 (3.99)	64.36 (3.70)	64.47 (3.95)
	Sex, Female, (N(%))	324 (60.9)	136 (50)	18 (41.9)
	HL dB per year	0.78	1.05	1.23
	Baseline PTA (dB HL)	11.51 (4.59)	25.38 (5.83)	52.80 (14.74)
	5-year PTA (dB HL)	13.30 (5.23)	29.14 (6.25)	56.04 (9.95)
	10-year PTA (dB HL)	19.29 (6.11)	35.88 (6.67)	65.09 (15.38)
	Baseline MMSE	28.97 (1.31)	28.59 (1.76)	28.02 (1.97)
	5-year MMSE	29.03 (1.56)	28.79 (1.79)	28.14 (2.01)
	10-year MMSE	28.03 (2.74)	27.72 (2.46)	26.45 (3.68)
Age 70 +	N=	258	274	66
Hearing Loss
	Age	74.35 (3.08)	75.75 (4.20)	78.08 (5.08)
	Sex, Female, (N(%))	150 (58.1)	162 (59.1)	42 (63.6)
	HL dB per year	1.05	1.32	1.24
	Baseline PTA (dB HL)	16.54 (5.15)	31.89 (6.11)	50.36 (10.30)
	5-year PTA (dB HL)	19.90 (5.95)	36.84 (5.65)	56.44 (8.87)
	10-year PTA (dB HL)	27.04 (7.17)	45.04 (8.02)	62.76 (18.13)
	Baseline MMSE	28.66 (1.60)	28.43 (1.62)	27.48 (3.17)
	5-year MMSE	28.33 (2.44)	28.25 (2.27)	27.94 (3.09)
	10-year MMSE	26.89 (3.56)	26.62 (3.70)	26.33 (2.86)

*Abbreviations: HL, Hearing loss; PTA, Pure-tone average; db, decibels; SD, Standard deviation; MMSE, Mini-Mental State Examination*.

**Table 3:** Descriptive statistics for cognitive function trajectory subgroups.

Sub-group	Characteristics	Normal	Low-Normal	Decline
		Mean (SD)	Mean (SD)	Mean (SD)
Age 55–69	N=	740	96	11
Cognitive
Function
	Age	62.59 (4.13)	63.66 (3.79)	61.91 (3.53)
	Sex, Female, (N(%))	436 (58.9)	38 (39.6)	4 (36.4)
	Baseline PTA (dB HL)	17.31 (10.67)	24.24 (17.61)	26.14 (18.28)
	5-year PTA (dB HL)	19.67 (11.47)	26.41 (16.50)	26.82 (17.12)
	10-year PTA (dB HL)	25.91 (12.53)	32.95 (19.53)	29.06 (28.58)
	Baseline MMSE	29.02 (1.21)	27.56 (2.17)	26.09 (3.75)
	5-year MMSE	29.40 (0.84)	25.94 (1.41)	21.64 (1.86)
	10-year MMSE	28.30 (2.09)	25.03 (3.35)	15.00 (5.96)
Age 70 +	N=	528	28	42
Cognitive
Function
	Age	75.26 (4.24)	76.45 (4.27)	76.5 (5.13)
	Sex, Female, (N(%))	315 (59.7)	16 (57.1)	23 (54.8)
	Baseline PTA (dB HL)	27.21 (12.51)	29.20 (14.6)	29.66 (12.65)
	5-year PTA (dB HL)	31.37 (13.22)	35.79 (13.22)	34.09 (14.47)
	10-year PTA (dB HL)	37.58 (14.75)	40.21 (8.99)	41.00 (14.29)
	Baseline MMSE	28.83 (1.19)	23.07 (2.62)	26.90 (2.05)
	5-year MMSE	28.80 (1.38)	27.69 (1.78)	21.38 (3.26)
	10-year MMSE	27.07 (3.03)	26.00 (3.55)	16.89 (4.20)

*Abbreviations: HL, Hearing loss; PTA, Pure-tone average; db, decibels; SD, Standard deviation; MMSE, Mini-Mental State Examination*.

### Hearing Loss and Cognitive Function Trajectories for the 70+ Years Age Group

Figure 1B shows the hearing loss and cognitive function trajectories for the older age group of 70+. Table 2 and Table 3 show the descriptive statistics for the hearing loss and cognitive function trajectories for this group. The trajectory classes were very similar to the trajectories identified for the younger age range of 55–69 years and labeled the same. Once again, there were age differences for the hearing loss trajectories (*F*_(2, 595)_ = 22.95, *p* < 0.001), with increasing age associated with greater hearing loss. However, there were no significant differences in the proportion of males and females for these trajectories. The rates of hearing loss for all three trajectories were also increasing over the 10-year period, an average rate of change in the three trajectories of 1.2 dB hearing loss per year. The mean (95% CI) bilateral hearing loss for the three trajectories were 21.3 (20.5–22.1), 38.1 (37.4–38.9), and 56.6 (55.0–58.3) dB HL. The three trajectories identified from GBTM for this age group had 42.7% belonging to the trajectory with no hearing loss, 45.4% belonged to a trajectory with mild hearing loss, and 11.9% of the group in the moderate to severe hearing loss trajectory. Similarly, there were three cognitive function trajectories labeled the same as the 55–69 age group. There were no significant differences in the proportion of males and females or in the ages of the three cognitive function trajectories. The mean (95% CI) MMSE scores for these three trajectories were 28.2 (28.1–28.4), 25.5 (24.8–26.2), and 21.6 (20.8–22.3). For this age group, 87.9% of this cohort belonged to the high-normal cognitive function trajectory, 7.3% in the low-normal cognitive function trajectory, and only 4.9% belonged to a trajectory with declining cognitive function. Once again, the rate of decline in the declining trajectory was also at 1.1 MMSE score per year.

### Dual Trajectory Models for Hearing Loss and Cognitive Function

**Figure 2:**
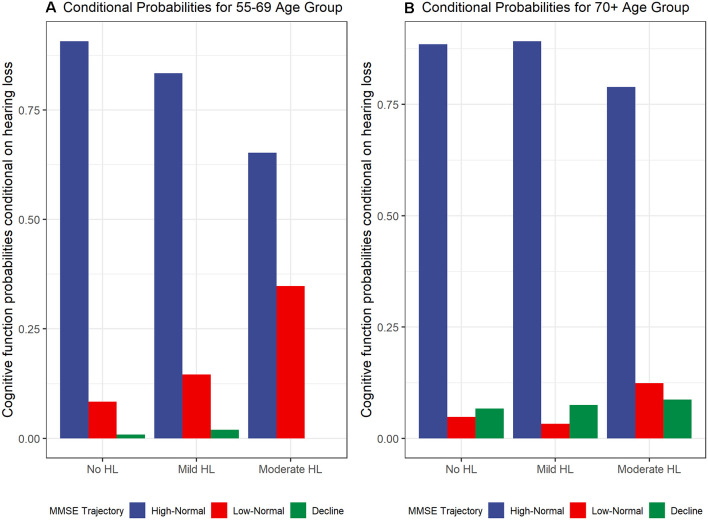
Cognitive function trajectory (MMSE) probabilities conditional on hearing loss trajectories for **(A)** 55–69 years age group and **(B)** 70+ years age group.

To examine the contemporaneous development of hearing loss and cognitive function dual-GBTMs were examined. The conditional probabilities of co-occurring developmental trajectories for hearing loss and cognitive function for the 55–69 age group are shown in Figure 2A. There was a 90.7% (*n* = 491) probability for those in the no hearing loss to also belong to the high-normal cognitive function trajectory. From the linkage probabilities, there is evidence for large overlap or co-movement between no hearing loss and mild hearing loss 83.4% (*n* = 219) and high-normal cognitive function. For no hearing loss there was an 8.4% (*n* = 44) probability of belonging to the low-normal trajectory and 14.6% (*n* = 40) probability for mild hearing loss. In contrast, those that belong to the moderate to severe hearing loss trajectory had only a 65.2% (*n* = 28) chance of belonging to the high-normal cognitive function trajectory with the remaining 34.8% (*n* = 15) probability of belonging to the trajectory with lower (but still within the normal range) cognitive function trajectory. The cognitive decline group was small and as mentioned earlier only accounted for 1.4% of the whole group. Those in the mild hearing loss trajectory had a 2% (*n* = 5) probability of belonging to this trajectory and those in the no hearing loss trajectory had a 0.9% (*n* = 5) chance of belonging to this trajectory.

For the 70+ group, the conditional probabilities are shown in Figure 2B. The probabilities for belonging to the high-normal cognitive function group were similar for the no hearing loss and mild hearing loss trajectories. The linkage probabilities for no hearing loss and high-normal cognitive function were also found to have a large overlap at 88.5% (*n* = 225). There was a larger probability for mild hearing loss trajectory to also belong to the high-normal cognitive function trajectory compared with those in the younger 55–69 age group at 89.2% (*n* = 248). There were smaller probabilities of belonging to low-normal cognitive trajectories compared to the younger age group at 4.8% (*n* = 12) for no hearing loss, 3.3% (*n* = 8) for mild hearing loss, and 12.4% (*n* = 8) for moderate hearing loss. There was an increasing conditional probability between levels of hearing loss trajectories and the cognitive decline trajectory with 6.7% (*n* = 20), 7.5% (*n* = 20), and 8.7% (*n* = 6) for no hearing loss, mild hearing loss, and moderate to severe hearing loss respectively.

### Hearing Aid Use With Cognitive Function Trajectories

The influence of hearing aid use on the cognitive function trajectories was also examined. We utilized a subgroup of hearing aid users and a matched comparison group from propensity score matching. For distribution of the propensity scores for the unmatched and matched subgroups see Supplementary Material for details. The SMD for the two groups was less than 0.2 for age (SMD = 0.15) and sex (SMD = 0.15), but greater than the 0.2 cut-off for PTA(dB HL; SMD = 0.33). The variance ratio was close to 1 (and below 2) for all three covariates, with age = 1.09, sex = 1.05, and *PTA*_(dB HL)_ = 1.14, showing acceptable matching. There was no significant difference for age (*F*_(1, 288)_ = 1.65, *p* = 0.2) or sex (*χ*^2^ = 1.71, *p* = 0.2), however, the hearing loss group remains significantly more severe in hearing loss from PTA(dB HL; *F*_(1, 288)_ = 8.43, *p* = 0.004) between the two subgroups. We tested for differences in the proportion of hearing aid users and non-hearing aid users for the three cognitive function trajectories While there was no significant difference in proportion between the two groups in the three cognitive function trajectories (*χ*^2^ = 4.0, *p* = 0.1), there was a greater percentage of hearing aid users in the high-normal cognitive function trajectory with 91.7% (*n* = 133) compared with 84.1% (*n* = 122). Fewer hearing aid users were in poorer cognitive function trajectories such as the low-normal trajectory [2.1% (*n* = 3) compared with 4.8% (*n* = 7)] and cognitive decline trajectory [6.2% (*n* = 9) compared with 11.0% (*n* = 16)]. Despite the higher severity of hearing loss in the hearing-aid users’ group, there were modest protective effects found from hearing aid use on cognitive functions.

## Discussion

This study examined the developmental pathways of two interrelated complications prevalent in the older population—hearing loss and cognitive impairment. We identified through the GBTM approach, three trajectory profiles with frequency distributions for both hearing loss and cognitive function in two older age groups (55–69 years and 70+ years). For hearing loss profiles, the trajectories were, no-hearing loss (62.8% and 42.7%), mild hearing loss (32.0% and 45.4%), and moderate to severe hearing loss (5.2% and 11.9%) for the two age groups, respectively. For the cognitive function profiles, the trajectories were, high-normal (86.9% and 87.9%), low-normal (11.7% and 7.3%), and declining cognitive function (1.4% and 4.9%), for the two age groups, respectively. Further, the findings have extended our understanding of hearing loss and cognitive impairment by examining the two complications as they co-occur. Dual-GBTM allowed for a comprehensive understanding of the underlying relationship between these two outcomes by examining the dynamic linkage between the concurrent trajectories (Xie et al., 2010). We found hearing loss and cognitive function trajectories to be co-occurring with a relationship between the increasing probability of belonging in the cognitive decline trajectory and the increasing levels of hearing loss.

Despite the known cognitive function differences that occur at 70 years and older (Harada et al., 2013), the trajectory profiles in this study were similar for the two age groups examined. Differences between the two age groups occurred in the distribution of older adults belonging to each of the profiles. Most of those 55–69 years belonged to the no hearing loss, whereas the 70+ age group had an almost equal number of no loss and mild hearing loss and a greater percentage of moderate to severe hearing loss. These trajectory breakdowns are similar to hearing loss prevalence that show one-third of adults with adults 61–70 years of age have a hearing loss (Mitchell et al., 2011; Walling and Dickson, 2012) and approximately one half with hearing loss for adults 70–80 years (Michels et al., 2019). There were no significant differences found for age or sex for the older 70+ age group for all six trajectories. However, differences in age and sex were found for the hearing loss and cognitive function trajectories with increasing age associated with greater hearing loss and a smaller proportion of females in the hearing loss and cognitive impairment trajectories. The smaller proportion of females with hearing loss is supported by other research, with sex hormones such as estrogen, thought to play a role in signaling pathways to modulate differences in hearing (Murphy and Gates, 1997; Shuster et al., 2019). However, we did not find support for greater susceptibility in cognitive impairments for females. We found a smaller proportion of females in the cognitive impairment trajectories for the 55–69 years age groups. It may be that this is the younger age group as other studies are finding greater cognitive impairment in females, especially the case after age 75 (Wang et al., 2020).

Surprisingly, the profile for high-normal cognitive function for the two age groups was very similar at 86.9% and 87.9%. This may be a limitation of the MMSE, whereby ceiling effects are common especially in normal or predementia stages (Trzepacz et al., 2015). However, the frequency distribution for the other two trajectories was different. There was more representation in the cognitive declining trajectory for the older age group of 70+. Although the cognitive decline has been reported to begin in midlife, it has been found to occur more often at higher ages (over 70 years; Aartsen et al., 2002), this is consistent with the trajectories in this study. The prevalence of dementia reported for Australia is similar with approximately 1% of cases in the age range of 65–74, increasing to 6% at 75–84 years of age (Australian Bureau of Statistics, 2018). Both the hearing loss and cognitive function trajectories from this study population are comparable with other research on these demographics demonstrating that GBTM has successfully identified the relevant trajectories for older adults.

Further, the results from this study also demonstrated that the different hearing loss profiles have an impact on the contemporaneous development of cognitive impairment. Dual trajectories were applied to examine the co-occurring pathways and showed that up to 90% probability of belonging in the high-normal cognitive function trajectory was conditional on belonging to the no hearing loss trajectory, irrespective of age group. The linkage probabilities show a high level of temporal correspondence between no hearing loss and normal cognitive function. Having a higher severity of hearing loss reduces the probability of belonging to a high-normal cognitive trajectory to 65–79%. This result strongly supports maintaining optimal normal hearing function for normal cognitive function. There was a relationship between severity of hearing loss and cognitive decline. Those belonging to the no hearing loss trajectory had a 6.5 probability of being in the cognitive declined trajectory and this increased with severity of hearing loss to 7.5% probability for mild hearing loss and 8.7% probability for moderate to severe hearing loss. These results demonstrate a steady decline in cognitive function for those belonging to increasing hearing loss severity trajectories. Similarly, compared to normal hearing, another research has found a two-, three- and five-fold increase in risk for dementia with mild, moderate, and severe hearing impairment (Lin et al., 2011b). There is evidence from other longitudinal studies that show hearing impairment to be independently associated with a 30–40% rate of accelerated cognitive decline (Lin and Albert, 2014). In this study, the average MMSE score for the cognitive declining trajectories was below the cut-off of 25, at 21.0 and 21.3 for both age groups, those in this trajectory have scores suggesting mild dementia (Creavin et al., 2016). Hearing loss has been found to be independently associated with incident all-cause dementia (Lin et al., 2011b). The results examining dual trajectories for hearing loss and cognitive function support the hypothesis for the underlying mechanisms for this association as reviewed in Lin and Albert (2014). With progressive damage to cochlear function from aging, cognitive load would always be a “dual-task” for people with hearing impairment thus adversely affecting cognitive performance and increasing risk of dementia.

It is of concern that hearing loss, although highly prevalent in older adults and prospectively associated with incident dementia, often remains untreated (Popelka et al., 1998; Lin et al., 2011b). The results of this study suggest that maintaining optimal hearing function is valuable for the maintenance of better cognitive function. We sought to examine whether there would be benefits on cognitive function from interventions that improve hearing function, through hearing aid use. We compared a hearing aid subgroup with a comparison group matched for age, sex, and audiometric measures of hearing loss. While we did not find any significant difference in proportions of hearing aid use vs. matched comparison group in the three cognitive function trajectories, we did find a consistently greater representation of non-hearing aid users in the trajectories with poorer cognitive function. There was also a greater proportion of hearing aid users in the high-normal cognitive function trajectory (91.7%) compared with non-hearing aid users (84.1%). The results suggest that hearing intervention may be protective against poorer cognitive function. However, the beneficial effects from this study are modest, and further investigation perhaps with an intervention is required to determine if there are mitigating effects from hearing aid use.

The strengths of this study include utilizing a modeling technique that allows for the identification of distinct heterogenous patterns within a large representative longitudinal dataset. In addition, we were able to explore developmental changes in these two common neurological complications for the older population as they evolve contemporaneously, showing their relationship and co-occurrence of movement with each other. As to limitations, we examined the trajectories as they were occurring without examining potential unmeasured confounders. We have tried to limit the confounding influence of demographic confounders such as age through stratification into subgroups and have also examined effects of sex on these trajectories, however, other potential unmeasured confounders, such as noise exposure (Huang et al., 2021), physical activity profiles (Kuo et al., 2021a), dietary intake (Gopinath et al., 2010), and birth weight (Gopinath et al., 2021) may influence these trajectories and need further exploration. Although we utilized a large representative sample, the sample size in each of the subgroups outside of the normal hearing and high-normal cognitive are modest and this should be noted. Also, the number of hearing aid users was small and mostly of moderate hearing loss, which is another limitation. We were unable to find any significant differences between the hearing aid sample and the matched comparison group, however, there was a trend that hearing aid use may be protective for cognitive functions. A larger sample size of hearing aid users would provide more power to detect differences especially within small trajectories such as cognitive decline. A larger hearing aid users’ sample would also allow for the examination of the data for level of hearing aid use which may provide better information for hearing intervention and cognitive function. Another limitation is that there were only two follow-up time points with a total of three time points for the model. This meant that only linear relationships could be explored. Other developmental relationships such as quadratic changes would require more than three time points, this may be of interest with hearing loss and cognitive function changes over time.

This study utilized a representative population-based sample of older adults in the Australian community. It identified trajectories in proportions that were in agreement with our current understanding of hearing loss and cognitive impairment in the older population worldwide, showing support for the generalizability of the trajectories. With the growing evidence that these two conditions are interrelated, this study directly examined the progression and developmental trajectories of these complications contemporaneously. This adds to the existing evidence-base as dual trajectories demonstrated co-occurrence in developmental changes in these two common neurological complications for the older population. Results demonstrate a high probability for normal cognitive function with optimal/normal hearing. Examining both hearing loss and cognitive function longitudinally allowed the heterogeneity of both complications to be addressed together.

## Data Availability Statement

The raw data supporting the conclusions of this article will be made available by the authors, without undue reservation.

## Ethics Statement

The studies involving human participants were reviewed and approved by The University of Sydney. The patients/participants provided their written informed consent to participate in this study.

## Author Contributions

YT, DT, and BG conceived the study. YT, DT, BG, CL, CM, and PM developed the study design. CL conducted the data wrangling. YT drafted the article and conducted the analysis. YT, DT, BG, and CL made important contributions to the interpretation of data. All authors contributed to the article and approved the submitted version.

## Conflict of Interest

The authors declare that the research was conducted in the absence of any commercial or financial relationships that could be construed as a potential conflict of interest.

## Publisher’s Note

All claims expressed in this article are solely those of the authors and do not necessarily represent those of their affiliated organizations, or those of the publisher, the editors and the reviewers. Any product that may be evaluated in this article, or claim that may be made by its manufacturer, is not guaranteed or endorsed by the publisher.

## Supplementary Materials

Supplementary Figure 1 Propensity score density distribution for hearing aid group (blue) and no hearing aid group (red) for unmatched (unadjusted sample) and after matching (adjusted sample).
