# Supplementary material for: Co-occurring Hearing Loss and Cognitive Decline in Older Adults: A Dual Group-Based Trajectory Modeling Approach
Source: Front Aging Neurosci. 2021 Dec 24;13:794787. doi: 10.3389/fnagi.2021.794787 (PMC8740280; doi:10.3389/fnagi.2021.794787)
Supplement: Supplementary file 1 [file Table_1.docx]

# Supplementary Material

Fit indices are for 1 to 5 class trajectories for bilateral hearing loss (>25 dB HL) and cognitive function based on MMSE scores. For the 55-69 cohort, based on the rate of change for the information criterion fits statistics with a high entropy and significant BLRT, a 3-class solution was determined to be the best solution for hearing loss trajectories in the 55-69 age group. For cognitive function, a 3-class solution was also found to have the best fit indices. For the 70+ cohort, based on the rate of change for the information criterion fits statistics with a high entropy and significant BLRT, a 4-class solution was determined to be a slightly better solution for hearing loss trajectories in the 70+ age group compared to the 3-class solution. However, as the fit indices were similar and more importantly interpretability as well as easier comparisons to 55-65 age group cohort, we decided to use a 3-class solution for hearing loss for this group also. For cognitive function, a 3-class solution was also found to have the best fit.

Supplementary Table 1: Fit indices for the 54-69 age group with 1-5 class trajectory models. Hearing loss is measured as bilateral (>25 dB) and cognitive function measured using MMSE

|  | AIC | BIC | Entropy | LMRT* | BLRT* |
| --- | --- | --- | --- | --- | --- |
| Age 54-69: Hearing loss | | | | | |
| 1-class | 23120 | 23162.7 | NA | NA | NA |
| 2-classes | 22049.5 | 22115.9 | 0.92 | 0.55 | <0.001 |
| 3-classes | 21278 | 21368.1 | 0.89 | 0.2 | <0.001 |
| 4-classes | 20989 | 21102.8 | 0.85 | 0.51 | <0.001 |
| 5-classes | 20661.9 | 20799.4 | 0.88 | 0.19 | <0.001 |
| Age 54-69: Cognitive function | | | | | |
| 1-class | 15515.2 | 15557.9 | NA | NA | NA |
| 2-classes | 15080.8 | 15147.2 | 0.94 | 0.07 | <0.001 |
| 3-classes | 14936.4 | 15026.5 | 0.94 | 0.32 | <0.001 |
| 4-classes | 14847.4 | 14961.2 | 0.84 | 0.02 | <0.001 |
| 5-classes | 14765.9 | 14903.4 | 0.86 | 0.45 | <0.001 |

*p-values shown.

Abbreviations: Akaike information criterion (AIC), Bayesian information criterion (BIC), Lo-Mendell-Rubin test (LMRT), bootstrap likelihood-ratio test (BLRT), Not applicable (NA)
