# Supplementary material for: Co-occurring Hearing Loss and Cognitive Decline in Older Adults: A Dual Group-Based Trajectory Modeling Approach
Source: Front Aging Neurosci. 2021 Dec 24;13:794787. doi: 10.3389/fnagi.2021.794787 (PMC8740280; doi:10.3389/fnagi.2021.794787)
Supplement: Supplementary file 2 [file Table_2.docx]

Supplementary Table 2: Fit indices for the 70+age group with 1-5 class trajectory models. Hearing loss measured using 4FAHL and cognitive function measured using MMSE

|  | AIC | BIC | Entropy | LMRT* | BLRT* |
| --- | --- | --- | --- | --- | --- |
| Age 54-69: Hearing loss | | | | | |
| 1-class | 15445.4 | 15485 | NA | NA | NA |
| 2-classes | 14894.5 | 14956 | 0.79 | 0.04 | <0.001 |
| 3-classes | 14578.7 | 14662.2 | 0.85 | 0.3 | <0.001 |
| 4-classes | 14359.9 | 14465.4 | 0.88 | 0.12 | <0.001 |
| 5-classes | 14262.5 | 14390 | 0.84 | 0.29 | <0.001 |
| Age 54-69: Cognitive function | | | | | |
| 1-class | 11019.6 | 11059.2 | NA | NA | NA |
| 2-classes | 10727.8 | 10789.3 | 0.96 | 0.03 | <0.001 |
| 3-classes | 10610.2 | 10693.7 | 0.95 | 0.58 | <0.001 |
| 4-classes | 10546.6 | 10652 | 0.88 | 0.03 | <0.001 |
| 5-classes | 10449.8 | 10577.2 | 0.91 | 0.84 | <0.001 |

*p-values shown.

Abbreviations: Akaike information criterion (AIC), Bayesian information criterion (BIC), Lo-Mendell-Rubin test (LMRT), bootstrap likelihood-ratio test (BLRT), Not applicable (NA)
