# Supplementary figures and images for: Co-occurring Hearing Loss and Cognitive Decline in Older Adults: A Dual Group-Based Trajectory Modeling Approach
Source: Front Aging Neurosci. 2021 Dec 24;13:794787. doi: 10.3389/fnagi.2021.794787 (PMC8740280; doi:10.3389/fnagi.2021.794787)

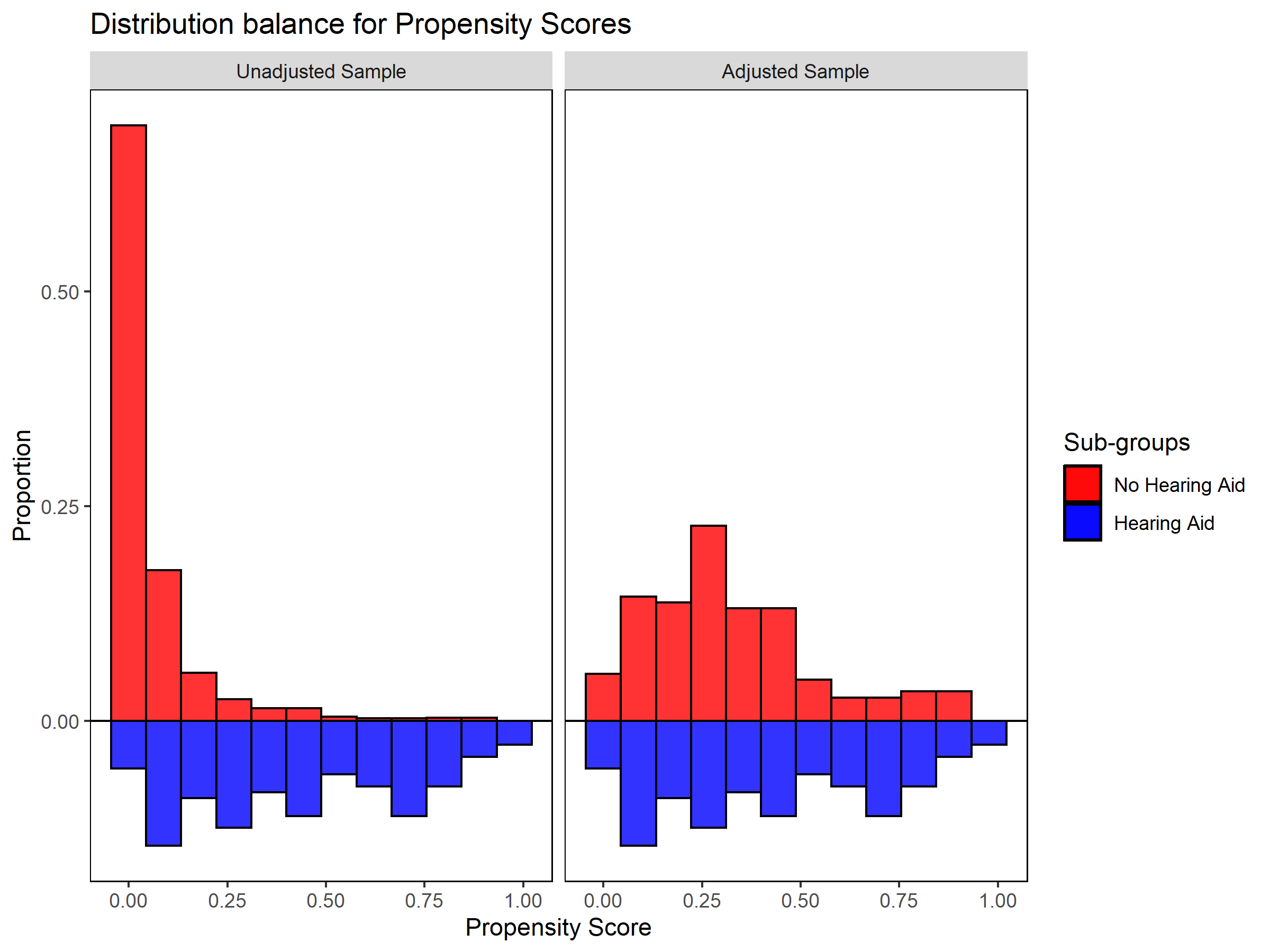

Supplement: Supplementary Figure 1 — Propensity score density distribution for hearing aid group (blue) and no hearing aid group (red) for unmatched (unadjusted sample) and after matching (adjusted sample). [file Image_1.TIF]
